# Robotic sentinel lymph node dissection experiences in endometrial cancer at our tertiary cancer treatment institution

**DOI:** 10.1590/1806-9282.20240696

**Published:** 2024-09-16

**Authors:** Erkan Şimşek, Sadık Gündüz, Özge Akdeniz Yıldız, Zinar Serhanoğlu, Levent Yaşar

**Affiliations:** 1 University of Health Sciences Turkey, Bakırköy Dr. Sadi Konuk Training and Research Hospital, Department of Obstetrics and Gynecology, Division of Gynecologic Oncology – İstanbul, Turkey.; 2 Kırıkhan State Hospital, Gynecology and Obstetrics Clinic – Antakya, Turkey.

**Keywords:** Sentinel lymph node, Endometrium cancer, Robot assisted surgery

## Abstract

**OBJECTIVE:**

In endometrial cancer surgery, sentinel lymph node dissection is used instead of staging surgery, particularly in advanced disease that is limited to the uterus. The aim of this study is to evaluate our practice of robotic sentinel lymph node dissection, which is applied to endometrial cancer patients in our tertiary cancer treatment center, according to the current literature, and to share our own data.

**METHODS:**

Included in our analysis are patients who underwent robotic sentinel lymph node dissection for endometrial cancer utilizing indocyanine green in our center between January 2018 and January 2024.

**RESULTS:**

In all, of the 93 endometrial carcinoma patients who underwent sentinel lymph node biopsy, 63 were classified as low-risk, while 30 were high-risk according to the European Society of Gynaecological Oncology and National Comprehensive Cancer Network guidelines. We found sentinel lymph nodes in both low-risk and high-risk patients, with an overall sensitivity of 96.32% (95% confidence interval [CI], 85.12–99.71), specificity of 100% (95%CI, 92.20–99.8), negative predictive value of 96.72% (95%CI, 87.03–99.89), and negative likelihood ratio of 0.06 (95%CI, 0.01–0.36).

**CONCLUSION:**

After evaluating our data retrospectively, we determined that we were compatible with the current literature.

## INTRODUCTION

Endometrial carcinoma (EC) is the most prevalent gynecologic cancer and the fourth most common malignancy among women in countries with high incomes^1^. In 2020, the world saw 417,367 new diagnoses and 97,370 new fatalities^2^.

Previously, treatment for EC was hysterectomy, bilateral sapingo-ophorectomy, and pelvic or pelvic and paraaortic lymph node dissection after surgery, according to the grade and pathology of cancer, both to determine the adjuvant treatment modality and to increase surveillance^3,4^. As a result of the latest studies^5–8^ in the literature and with the recommendation of NCCN guidelines 2023 (National Comprehensive Cancer Network)^8^, hysterectomy, bilateral salpingo-oophorectomy, and sentinel lymph node dissection (SLND) are now required in EC in all endometrioid grades (grade 1-2-3) and non-endometrioid pathology results for surgical treatment, and in the presence of a cancer determined to be limited to the uterus only in imaging methods.

In the treatment of EC, the evaluation of sentinel lymph node (SLN) is thought to be more appropriate in terms of the disease's progression than the evaluation of pelvic and paraaortic lymph nodes^8^. SLND is a less invasive technique for finding occult metastases in normal-appearing lymph nodes while avoiding full pelvic lymph node surgery^9^. When compared with full lymphadenectomy, this method detects more metastasis with fewer morbidities, such as intraoperative neurovascular damage or postoperative lymphedema^10^.

The Department of Gynecologic Oncology at Bakırköy Dr. Sadi Konuk Research and Training Hospital, Istanbul, Turkey, is a tertiary referral center for malignant gynecological surgery. Robotic surgery (Da Vinci Xi robotic system^®^, Intuitive Surgical Inc.) has been used since 2015 and is the preferred surgical approach for women with endometrial cancer unless contraindicated by uterine size, suspected disseminated disease, or anesthesia reasons.

The primary goal of this study is to share our findings on SLND with the literature. The other goal is to compare our own practice and experiences on this topic to the literature.

## METHODS

The study is a retrospective and single-center investigation. Patients who underwent robotic surgery for endometrial cancer in our center between January 2018 and January 2024 were included in our study. In these patients, SLND was attempted using ICG (indocyanine green).

Before robotic surgery, our patients’ malignancies were required to be limited to the endometrium only; the size of the uterus should be suitable for vaginal removal; the patient should be in the maximum Trendelenburg position suitable for robotic surgery; and the lungs should be able to handle the carbon dioxide pressure for anesthesia.

By ruling out extrauterine sickness, high-risk tumors underwent further imaging exams (computed tomography, magnetic resonance imaging, or fluorine-18 fluorodeoxyglucose positron emission tomography/computed tomography). Women with high-risk endometrial cancer (non-endometrioid histology, FIGO (The International Federation of Gynecology and Obstetrics) Grade 3, non-diploid flow cytometry, myometrial invasion greater than 50%, or cervical invasion) were scheduled for a complete pelvic and infrarenal paraaortic lymphadenectomy after an initial removal of SLNs. Furthermore, an infracolic omentectomy was performed in cases of non-endometrioid histology. Patients without any high-risk factors were allocated to the SLN biopsy. Following the NCCN 2023 guidelines, only SLND and suspicious bulky lymph node dissection are performed in all pathologies limited to the uterus.

A 25-mg vial of ICG powder was diluted in 20 mL of sterile water, and 4 mL of the solution was injected into the cervix at 3 and 9 o'clock locations, with 1 mL deep (1 cm) and 1 mL superficial (3–4 mm). The dye was administered slowly, at a pace of 10 s per location. The endoscopic systems compatible with indocyanine green were used in the da Vinci Xi robotic platform (Intuitive Surgical, California) for robotic surgeries. Mapping and dissection of the SLN were performed by experienced gynecologist oncologists working in our European Society of Gynaecological Oncology (ESGO) accredited center, as described in the NCCN guidelines.

The institutional review board (University of Health Sciences, Bakırköy Dr. Sadi Konuk Education and Research Hospital, Turkey, August 8, 2022) approved the study (Approval no. 2022/258).

The algorithm's performance was measured using sensitivity, false negative rate, and negative predictive value. It is impossible to report specificity, positive predictive value, or false positive rate because all positive SLNs must also be positive for lymph node metastasis (LNM). True positivity in LNM patients was characterized by a positive SLN or algorithm. A chi-square test was used to compare categorical variables. The descriptive information was given using the median (min–max) for continuous variables and the frequency (%) for categorical variables. p-values<0.05 were considered statistically significant. Statistical analyses were conducted using Stata 9.0 (StataCorp, College Station, TX).

## RESULTS

In all, of the 93 EC patients who underwent SLN biopsy, 63 were classified as low-risk, while 30 were high-risk, according to the ESGO and NCCN guidelines. There were no complications following the ICG injection, nor were there any surgical complications associated with the SLN biopsy. The average surgery duration from placing the robotic trocar to skin closure was 121 min (range 95–245). Median blood loss was 60 (range 40–150) mL with no blood transfusions administered, and two-thirds of the patients had postoperative hospital stays of 2 days.

According to our records and the surgical process, the anesthesiologist determined that three of our patients were not suited for robotic surgery. It was stated that the lung capacities of three of our patients were not suitable for robotic surgery because their chronic obstructive pulmonary disease and BMI (body mass index) were>45. Table 1 summarizes demographic information for the study population.

**Table 1:** Demographic and clinical characteristics.

	Median			Range
Age (years)	62			36–80
Body mass index (kg/m^2^) (BMI)	31			27–45
Hospital stay in days after surgery	2			1–7
Operation time (min)	121			95–245
Blood loss (mL)	60			40–150
Tumor size (mm)	8			6–80
**Depth of myometrial invasion**				
	**N: 93**			**Percentage**
No infiltration	3			3
<%50	66			70
>%50	22			25
Presence of lymphovascular space invasion	18			19
**Histology**				
	**N: 93**		**Percentage**	
Endometrioid	68		73	
Clear cell	9		9.6	
Serous	6		6.4	
Mix type (squamous adenocarcinoma)	8		7	
Carcinosarcoma	2		2	
Müsinöz adenoca	2		2	
**Sentinel lymph node detection (SLND) rates according to risk and body mass index**				
**Risk of endometrium carcinoma**	**N: 93**	**Percentage**	**Number of SLND**	Percentage
Low risk (FIGO Grade 1–2 endometrioid type)	63	67	65	100
Hıgh risk (FIGO Grade 3, non endometrioid type) (Two clear cell cases occurred from the polyp base Five endometrioid Ca were Grade 3)	30	33	28	93
**Patients body mass index**	**Number of patients**	**Percentage**	**Number of SLND**	Percentage
Body mass index<31	55	59	55	100
Body mass index>31	38	41	36	95
Total	93	100	91	97.8

Sentinel lymph nodes were identified in 91 out of 93 patients, resulting in a 97.8% total detection rate. Of the 91 patients with at least one SLN discovered, 89 had pelvic SLNs, resulting in a 97.8% detection rate. The detection rate was 87% (81 cases) for bilateral pelvic SLNs and 10.7% (10 cases) for unilateral pelvic SLNs, with six and four in the left and right hemipelvis, respectively. In four patients, there were three presacral and one internal iliac SLNs along with pelvic SLNs. In addition, by re-injecting ICG 10 min later, we were able to identify the SLN in two of our patients that we could not identify unilaterally after the first ICG injection.

After analyzing the distribution of anatomical places where SLNs were found, we discovered that it was 50% in the right and left obturatory areas, 47% in the right, and 48% in the left in both external iliac artery locations. Table 2 illustrates our detection rates for SLN, their anatomical locations, and metastatic lymph nodes.

**Table 2:** Sentinel lymph node (SLN) characteristics.

Detection rates of SLN	N: 93		Percentage	
Overall	91		97.8	
Bilaterally	81		87	
Unilaterally	10		10.9	
Undetected	2		2.1	
Anatomical location of SLNs in endometrial cancer				
Site of sentinel lymph node localization	Left	Percentage	Right	Percentage
Obturator area	41	50	42	50
External iliac area	39	48	40	47
Presacral area	1	2	2	3
Common iliac area	1		-	
The distribution of risk categories and corresponding rates of sentinel lymph node metastatic disease				
Positive metastatic SLN	Number of patients	Percentage	Number of metastatic lymph node	Percentage
Low risk (FIGO Grade 1–2 endometrioid type)	63	68	4	6
High risk (FIGO Grade 3, non endometrioid type) (Two clear cell cases occurred from the polyp base Five endometrioid Ca were Grade 3)	30	32	10	34
Total	93	100	14	15

Among the 63 patients with low risk, 65 had SLNs detected, and 4 had positive SLNs [3 micrometastasis and 1 isole tumor cell (ITC)] (6%). The SLN detection rate was 100% in this group. In the high-risk group, the overall SLN detection rate was 93% (28 cases), and all patients received a complete lymphadenectomy. SLNs were positive in 35.7% (10/28) of cases. The number of cases with positive SLNs in the last pathology report was defined as 60% (6/10) macrometastasis and 40% (4/10) micrometastasis. One of every two patients in whom we were unable to detect an SLN had clear cell carcinoma, whereas the other had grade 3 EC. Both patients had metastatic pelvic lymph nodes. Among patients with no pelvic nodes, 1.3% (1/77) experienced isolated paraaortic lymph node metastasis. Details of patients with metastatic SLN are shown in Table 3.

**Table 3:** Characteristics of patients with sentinel lymph node metastasis.

No.	Age	Histology	Pre-operative grade	Post-operative grade	Myometrial invasion	SLN	LND in algorithm steps	Other LNM sites
1	47	Endometrioid	1	2	>50%	Isole Tumor cell	Bilateral Pelvic	None
2	54	Endometrioid	2	2	<50%	Micrometastasis	Bilateral Pelvic	None
3	59	Endometrioid	2	3	>50%	Micrometastasis	Bilateral Pelvic + Paraaortic	Pelvic 3/14 positive
4	49	Endometrioid	2	3	>50%	Micrometastasis	Bilateral Pelvic+ Paraaortic	Pelvic 1/12 positive
5	62	Endometrioid	2	3	>50%	Micrometastasis	Bilateral Pelvic+ Paraaortic	Pelvic 2/11, paraaortic 2/14 positive
6	58	Endometrioid	3	3	>50%	Micrometastasis	Bilateral Pelvic+ Paraaortic	Pelvic 3/11, positive
7	65	Endometrioid	3	3	>50%	Macrometastasis	Bilateral Pelvic+ Paraaortic	Pelvic 3/16, paraaortic 1/7 positive
8	49	Endometrioid	3	3	>50%	Macrometastasis	Bilateral Pelvic+ Paraaortic	Pelvic 1/8 positive
9	67	Endometrioid	3	3	>50%	Macrometastasis	Bilateral Pelvic+ Paraaortic	Pelvic 3/11 positive
10	65	Serous	3	3	>50%	Macrometastasis	Bilateral Pelvic+ Paraaortic	Pelvic 3/15, paraaortic 1/9 positive
11	58	Serous	3	3	>50%	Macrometastasis	Bilateral Pelvic+ Paraaortic	Pelvic 2/9 positive
12	62	Serous	3	3	<50%	Micrometastasis	Bilateral Pelvic+ Paraaortic	Pelvic 3/14 positive
13	55	Clear cell	3	3	<50%	Macrometastasis	Bilateral Pelvic+ Paraaortic	Pelvic 2/11 positive
14	65	Clear cell	3	3	>50%	Macrometastasis	Bilateral Pelvic+ Paraaortic	Pelvic 2/10 positive
15	56	Endometrioid	3	3	>50%	None	Bilateral Pelvic+ Paraaortic	Paraaortic 2/8 positive

Note: SLN: sentinel lymph node; LND: lymph node dissection; LNM: lymph node metastasis.

The majority of the patients with SLN positive (10/14, 72%) are in advanced stages. In addition, isolated tumor cells were found in only one patient at an early stage. The histopathologic outcomes of SLNs and nodes obtained during systematic lymphadenectomy were compared. We found SLNs in both low-risk and high-risk patients, with an overall sensitivity of 96.32% (95%CI, 85.12–99.71), specificity of 100% (95%CI, 92.20–99.8), negative predictive value of 96.72% (95%CI, 87.03–99.89), and negative likelihood ratio of 0.06 (95%CI, 0.01–0.36).

## DISCUSSION

We conducted this study to discuss our own experiences and compare our findings to the existing literature on the robotic-assisted sentinel lymph determination technique utilizing endometrial treatment (ICG). Our SLND rates were similar to those reported in the current literature^11,12^. It was observed that our bilateral SLND rates, which especially increase the success of SLND, were similar to the literature^13,14^. Our false-negative SLN evaluation rate, which is essentially the most crucial marker that changes the patient's stage, was determined to be 1.3% (one patient). This rate is within the boundaries established in the literature^6^.

Patients were separated into two groups based on their BMI: <31 and >31, with 41% (38 patients) falling into the second group. The group also includes two (2%) patients in whom we could not detect SLNs. Previous research has suggested that the difficulty in finding SLNs in obese patients stems from both the difficulties of anatomical dissection and the high risk of bleeding during dissection^15,16^.

It has been reported that systemic lymphadenectomy does not reduce lymphatic metastatic recurrence in the course of the disease after surgery^17^. Our negative predictive value for sentinel lymph node discovery was 97.8%, indicating that sentinel lymph node dissection can fully replace systemic lymphadenectomy.

We predicted that we have rates close to those in the literature due to the fact that we use only ICG as a dye to detect sentinel lymph nodes and that the possible SLN is removed after determining the afferent and efferent lymphatic channels with robotic surgery^18^. We believe that watching the lymphatic channels during surgery and entering the anatomical locations where the SLN can be located increases the chances of successfully finding the sentinel lymph node, particularly in obese individuals. Following the literature's guidance^19^, we were able to find the unilateral SLN in two of our patients who had previously gone undetected by reinjecting 10 min later.

Our SLND rates in early and late-stage endometrial cancer are consistent with the literature^7–20^. After evaluating the anatomical locations of the SLNs we determined, we determined that we reached the locations and rates previously stated in the literature^10–18^. The average surgery time for robotic surgery and SLND, the amount of bleeding, and the patient's hospital stay are consistent with previous research findings^21^.

During robotic SLN sampling, there were patients who were obese, particularly those with low lung capacity (three patients), in the maximum Trendelenburg position at a slope of 32%, and due to the CO_2_ pressure in the abdomen, we had difficulty during surgery and were unable to perform it. In our clinic, we perform SLND surgeries using laparoscopic and VNOTES (Vaginal Natural Orifice Transluminal Endoscopic Surgery) techniques for our patients with endometrial cancer. We performed operations on our patients with high BMI and chronic obstructive pulmonary disease without any problems with anesthesia. We have experienced the accuracy of the opinions in the most recently published study^22^. We intend to report on these experiences in a separate study.

While the limitations of our study are that it is retrospective and the number of cases is low, the fact that it is single-centered, performed by the same surgical team, and our SLN detection rates and anatomical locations are similar to those in the literature indicate the strength of our study.

## CONCLUSION

Sentinel lymph node dissection in endometrial cancer is about to become the mainstay of surgical treatment in a disease limited to the uterus. It is pleasing for us to have data similar to the literature in this process.
